# The experiences and impact of the COVID-19 pandemic on young carers: practice implications and planning for future health emergencies

**DOI:** 10.1186/s13034-023-00697-6

**Published:** 2024-01-03

**Authors:** D Hayes, D Fancourt, A Burton

**Affiliations:** The Social Biobehavioural Research Group, Research Department of Behavioural Science and Health, Institute of Epidemiology & Health Care, University College London (UCL), 1-19 Torrington Place, London, WC1E 7HB UK

**Keywords:** Mental health, Wellbeing, COVID-19, Qualitative, Young carers, Health emergencies

## Abstract

**Background:**

Young carers are children or young people aged up to 25 years old who undertake unpaid caring responsibilities for a friend or family member. Young carers faced significant challenges brought on by the COVID-19 pandemic. We explored the impact of the pandemic and associated restrictions on mental health, wellbeing and access to support in young carers in the United Kingdom (UK) to understand how to improve services, as well as support this population in future health emergencies.

**Method:**

We conducted 22 qualitative semi-structured interviews from May to November 2021 with 14 young carers and eight staff working in organisations that supported them. Interviews took place remotely over video or telephone call and explored participant experiences of the pandemic and its impact on their health, wellbeing and caring responsibilities. We used reflexive thematic analysis to analyse interview transcripts.

**Results:**

We identified four overarching themes pertaining to the impact of the pandemic and associated restrictions on mental health, wellbeing and access to support in young carers in the UK: (1) challenges in protecting loved ones from the virus, (2) changes to and loss of routine, (3) reduced access to pre-pandemic informal and formal support structures and (4) better understanding of inner resilience and goals. Many participants struggled with their mental health and wellbeing as a result of pandemic related restrictions which impacted on support structures for themselves and the individual they cared for. However, positive impacts pertained to additional support provided by local authority and third sector organisations.

**Conclusions:**

Our findings highlight some of the changes that affected young carers during the COVID-19 pandemic. The impact of changes to routine and a reduction in pre-pandemic support were the greatest concerns reported by participants in this study. The additional support provided by local authority and third sector organisations during social restrictions suggests such organisations could play a greater role in supporting this population going forward and that schools and Governments may wish to put in additional strategies and provisions to protect young carers in the future.

**Supplementary Information:**

The online version contains supplementary material available at 10.1186/s13034-023-00697-6.

## Background

Prevalence studies across Europe highlight that 7–8% of children and young people aged 25 and under undertake caring responsibilities [1–5]. In the United Kingdom (UK), there are more than one million young people who are classified as ‘young carers’ [5, 6]. These individuals provide substantial care-giving assistance to a family member or friend with a physical or mental illness, disability or substance misuse problem [7, 8]. Referred to as a ‘hidden army’ [9–11], young carers take on multiple and varied responsibilities, including delivering personal care, support with activities of daily childcare and assistance with healthcare appointments [11–13]. However, the work of young carers is often invisible to statutory organisations [14], meaning that their needs are not met on a day-to-day basis and even less so during national and international health emergencies.

Estimates suggest that the majority of young carers are in their early teenage years [15]. This age is a time of important biological, psychological and social change for the individual as they enter adolescence and become a young adult [16, 17]. For most young people, adolescence is marked by forming closer social connections to their peers, developing their own interests and sense of identity and starting on a path of navigating their own independence [18]. This is often not the case for many young carers, who, by virtue of their role are unable to share and engage in these experiences to the same degree as their non-carer peers [8]. This too can extend into their early adult years as caring responsibilities can influence their ability to move into higher education or engage with particular employment opportunities such as full time work or work involving travel [8].

Caring for a loved one can also significantly impact mental health and wellbeing; around half of young carers report stress and mental health difficulties associated with caring [16]. In a meta-analysis, children and young people with chronically ill parents in need of caregiving were more likely to have high rates of both internal and external behavioural problems compared to those without caring responsibilities. This effect was increased when the family unit was of lower socio-economic status, the illness was longer in duration or when the child or young person was younger in age [19]. Moreover, externalising difficulties were greater when a higher proportion of mothers were unwell and when the young carer was supporting a single parent [19]. These difficulties may persist into adulthood, with young carers more likely to be hospitalised due to their mental health difficulties and to report feelings of suicidal ideation [20].

### Young carers during the COVID-19 pandemic

The COVID-19 pandemic disproportionally affected young people’s mental health compared with other age groups [21]. Specifically for children and young people, higher rates of depressive symptoms and lower rates of life satisfaction were reported during the pandemic compared to a matched control the year before [22]. Reasons for these changes included educational disruption and school closure affecting routines and the ability to interact and socialise with peers [23, 24], increased uncertainty about the future at a time of key developmental milestones [25] and an increase in external stressors, including bereavement [26]. Research exploring the impact of the pandemic on young carers suggests poorer mental health and wellbeing outcomes compared to those without caring responsibilities. For example, a longitudinal survey in Italy observed higher rates of mental health difficulties in young carers compared to young people without caring responsibilities including higher anxiety and depression and lower wellbeing. Higher levels of COVID-19 specific outcomes were also observed such as risky health behaviours, loneliness, home violence and fear of COVID-19 [27]. Similarly, data from the Millennium Cohort study in the UK showed that young carers had higher psychological distress and lower mental wellbeing than their non-carer counterparts during the early stages of the pandemic [28]. Psychosocial risk factors including poor quality sleep, low social support and increased feelings of loneliness largely explained the findings.

Qualitative findings also lend support to the role of psychosocial risk factors during COVID-19 and shed further light on the impact of the pandemic on children and young people with caring responsibilities. For example, a rapid approach, mixed-methods study identified that during the pandemic, support for young carers was withdrawn from external providers as well as friends and family, often leaving young carers overwhelmed, unable to cope and without coping mechanisms and routines [29, 30]. The loss of school as a sanctuary and source of support was highlighted, as well as not being able to have time and space to manage their mental health difficulties. Other studies have identified similar themes [10, 31], but have also reported positive experiences associated with the COVID-19 pandemic, including more time for self-care and more time with family [10, 31].

## This study

Young carers are referred to as a ‘hidden army’ and are often invisible to statutory organisations. This means that not only are their physical and psychological needs frequently not met in day-to-day life, but that in times of emergency, the exacerbation of these needs can be overlooked. Qualitative research on this population and the COVID-19 pandemic has mostly focused on the initial and early stages of the pandemic [10, 29–31], is often limited to very small sample sizes [10, 31] and only focuses on the views of the young carers [31] rather than sourcing multiple stakeholder views, such as those running services. This study, therefore, explored the experiences of young carers and those providing young carer services during a global health emergency, with the aim of developing strategies for the protection of, and support for young carers during future health emergencies.

## Methods

### Design

A qualitative interview study was conducted to explore the impact of the COVID-19 pandemic and associated restrictions on mental health, wellbeing and access to support in young carers aged 13 to 24. The work forms part of the University College London COVID Social Study.

### Participants

Participants were recruited using convenience sampling via national and local third sector organisations providing services and support to young carers, social media and via a study newsletter. Both young carers aged between 13 and 24 years old and staff working for organisations that supported young carers were eligible to take part in the study. Participants also had to be based in the UK and be able to provide informed consent or assent alongside parent/guardian informed consent if aged 13–15 years old. Study posters containing researcher contact details were sent to UK based organisations providing services to young carers for distribution to their staff and young carers via newsletter/mailing lists. A researcher AB also attended online and in-person (when restrictions allowed) young carer support groups provided by these organisations to introduce the study and answer questions about the research. For those that participated in an interview, a £10 voucher was provided for their time.

### Data collection

**Fig. 1:**
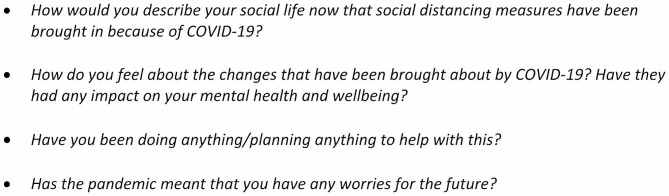
Young carer topic guide question examples

Interviews took place between May and November 2021. Semi-structured topic guides adapted from previous interviews with different populations [24, 32]were used to conduct the interviews (See Fig. 1 for example questions). For young carers, questions explored the impact of the COVID-19 pandemic and associated restrictions on mental health, wellbeing and access to support. For staff, questions included the impact of the COVID-19 pandemic and associated restrictions on service user mental health and wellbeing, challenges to operational practices and service delivery and service adaptations. Interviews were conducted remotely either via telephone or Microsoft Teams. Participants were interviewed alone with no other person present in the room and no follow up interviews were carried out. The interviewer had no prior relationship with any of the participants. All interviews were conducted by AB, a female, Senior Research Fellow with a PhD and substantial experience in qualitative research methods and mental health research. All procedures involving human participants were approved by the UCL Ethics Committee (project IDs: 14895/005 and 6357/002).

### Data analysis

Audio files were transcribed verbatim by an external transcription company approved by UCL. Personal and identifiable data were removed from transcripts before analysis to maintain confidentiality. Transcripts were not returned to participants for comment or correction. Transcripts were then imported into NVivo 12 [33] for data management and subsequent coding. A reflexive thematic analysis was conducted [34]. First, initial codes were developed based on the topics explored in the interview schedule. Codes were then applied to relevant fragments of text within the transcripts, however new codes were also developed when passages of participant accounts did not align with the pre-determined codes. Coding was undertaken primarily by DH with additional coding support provided by AB. The codes were discussed and refined during the data analysis period within regular meetings between the research team. Codes were then organised into groups of similar constructs and labelled with overarching theme and subtheme names, with DH presenting findings to the research team for discussion and refinement at two follow up meetings. Revisions to findings were made based on feedback at these meetings and a final set of themes and sub themes were agreed. Findings were not presented to participants.

### Trustworthiness of the research

Four of Shenton’s (2004) techniques were followed to ensure research credibility [35]. First, to encourage honest responses, the interviewer AB built a rapport with participants prior to data collection by meeting with them to explain the purpose of the study, allowing time for questions, reminding participants that there were no right or wrong answers, encouraging them to share as much or as little as they felt comfortable with and explaining that their responses would be pseudonymised. Second, feedback sessions were held between the primary coder DH the senior researcher AB and another qualitative researcher during the analysis phase AMK to develop ideas and possible interpretations of the data. Third, a reflexive log was kept by the interviewer and primary coder. Lastly, the interviewer told participants about their research background and reasons for conducting the research prior to the interview.

## Results

14 young carers and eight staff working for young carer organisations participated in an interview for this study. Interviews with young carers ranged in length from 37 to 69 min (*M = 52, SD 11).* The mean age of young carer participants was 19 years old and the majority were female (71%) and lived with family (93%). The largest proportion of young carers identified as ‘Asian Bangladeshi’ (43%) and were at school or university (36%). Parents were the most frequent recipient of care from the young person (43%).

The mean age of service providers was 39 years old with, on average, seven years of experience working with young carers. Interviews with service providers ranged in length between 36 and 66 min (*M = 48, SD 13)*. All identified as White and outlined that the services they provided included advice and information, as well as signposting to services.

Demographics of participants are presented in Tables 1 and 2.

**Table 1:** Young carer characteristics

Characteristic	Mean (range)/ n(%)
**Age**	19 (14–24 years old)
**Gender**	
Female	10 (71%)
**Ethnicity**	
Asian Bangladeshi	6 (43%)
White British	3 (21%)
Asian Pakistani	2 (14%)
Mixed Race – White and Black/Black British – Caribbean	1 (7%)
Black/Black British – African	1 (7%)
White other	1 (7%)
**Employment status**	
At school/university	5 (36%)
At school/university and part time employment	4 (29%)
Part time employed	3 (21%)
Full time employed	1 (7%)
Unemployed and seeking work	1 (7%)
**Living situation**	
With family	13 (93%)
Live alone	1 (7%)
**Who they provide care to**	
Parent	6 (43%)
Grandparent	3 (23%)
Multiple family members	3 (23%)
Sibling	1 (7%)
Friend	1 (7%)

**Table 2:** Service provider characteristics

Characteristic	Mean (range)/ n(%)
**Age**	39 (22–53 years old)
**Gender**	
Female	6 (75%)
**Ethnicity**	
White British	6 (86%)
White Scottish	1 (7%)
White Other	1 (7%)
**Number of years working with Young Carers**	7 (1–23 years)
**Client group**	
Young adult carers (aged 18–24)	3 (38%)
Young Carers (aged < 18) and young adult carers	3 (38%)
Young Carers (aged < 18)	2 (25%)
**Services provided**	
Advice and information	8 (100%)
Signposting to other services	8 (100%)
Essential items (food/hygiene products)	7 (88%)
Psychological or pharmacological treatments	3 (38%)

### Themes

We identified four overarching themes pertaining to the impact of the pandemic and associated restrictions on mental health, wellbeing and access to support for young carers in the UK: (1) challenges in protecting loved ones from the virus, (2) changes to and loss of routine, (3) reduced access to pre-pandemic informal and formal support structures and (4) better understanding of inner resilience and goals. Themes and sub themes are illustrated in Table 3. and described in detail below with supporting quotations from participants.

**Table 3:** Themes and subthemes

Theme	Subtheme
1. **Challenges in protecting loved ones from the virus**	1.1. **Confusing and rapidly changing advice**
	1.2. **Outsiders flouting the rules**
	1.3. **Difficulties encouraging loved ones to follow the rules**
2. **Changes to and loss of routine**	2.1. **Loss of routine afforded via education**
	2.2. **Increased caring responsibilities due to lack of support**
	2.3. **Loss of self-care routines and practices**
3. **Reduced access to pre-pandemic informal and formal support structures**	3.1. **Limited in-person contact with the wider family**
	3.2. **Limited contact with friends for emotional support**
	3.3. **Limited wellbeing support via educational pathways**
	3.4. **Limited health and social care services to support young carers**
	3.5. **Local authority and non-statutory bodies ‘filling the gap’**
4. **Better understanding of inner resilience and goals**	

### Theme 1: challenges in protecting loved ones from the virus

Young carers outlined various challenges when it came to protecting themselves and ultimately their loved ones from catching the virus. The main challenges identified by participants included rapidly changing government advice, other individuals not following the rules and difficulties persuading loved ones to follow the rules.

### Confusing and rapidly changing advice

The majority of young carers described the importance of following the guidelines with the understanding that they were put in place to protect themselves and the public by limiting exposure and transmission of the virus. In line with this, many young carers attempted to follow Government advice and take precautions such as self-isolating, forming support bubbles, getting vaccinated, social distancing and wearing masks.

*“I did self-isolate and it was to protect both myself and my mum from the virus and I didn’t want to be a carrier and then spread it” (Young carer nine)*.

For some, taking these precautions was intrinsically linked to their role as a young carer, where they felt a strong sense of duty to protect the person they were looking after due to them having an increased vulnerability to the virus.

*“I feel as though if I wasn’t a carer, I probably wouldn’t think of the guidelines as something quite serious, but because I am, I have to think about not only myself, but the people I’m caring for.”” (Young carer one)*.

Despite the general consensus that following the guidelines was a good idea, some young carers reported that they lacked clarity or sense and were therefore difficult to follow:

*“In terms of the tiers, it was a bit all over the place,, it was quite unnerving, because we weren’t sure where we stood” (Young carer seven)*.

This left young carers feeling confused, frustrated or anxious, as at times, there was discordance between official guidelines and how they felt they were supposed to act to help stop the spread of the virus and protect themselves and others.

*“Even though social distancing and everything has eased… I need to maintain my distance, just so we’re not spreading anything” (Young carer three)*.

### Outsiders flouting the rules

Some young carers reported tensions with others who they perceived as not being diligent or stringent in following the guidance. In most cases this pertained to members of the general public but also to acquaintances and friends, particularly at school. Young carers felt let down by others and perceived their actions as selfish and putting vulnerable people at risk:

*“I [felt] quite a lot of annoyance, particularly at people. They just haven’t properly thought about what they’re doing and how it can impact people” (Young carer 14)*.

In the latter stages of the pandemic, as young carers returned to in person lessons, school was often a place of tension for them as they perceived their peers as lax with following the guidelines. There were particular concerns around mask wearing where often masks were pulled down, partially covered their peers faces, or were not worn at all:

*“There are some people [at school] who don’t always wear their masks, as in pull them down a bit. I don’t with mine, except to drink” (Young carer 12)*.

Service providers also reported hearing concerns from young carers around peer non-conformity to the guidelines when attending school and the anxiety that resulted from this:

*“[They] were anxious at going to school where people weren’t socially distancing” (Service provider seven)*.

Sticking to the rules when others were not, both inside and outside of school settings sometimes resulted in young carers feeling sad because they were missing out on opportunities to have the same enjoyable social experiences as their peers:

*“I’ve seen people outside [the house]…they look like they’re having a fun time, and there is a risk that they could catch Coronavirus, at least they are taking the approach that if I go, I go happy, rather than being inside, miserable.” (Young carer seven)*.

### Difficulties encouraging loved ones to follow the rules

Whilst it was more often perceived that people outside of the immediate family unit were less likely to follow the rules and guidelines, some young carers also discussed how their family members found abiding by the guidelines a challenge. When this related to the individual being supported by the young carer, they were often unable to follow the guidelines due to a lack of cognitive capacity, or because of mental health difficulties:

*“I think she does need some guidance and support, especially when it comes to wearing a mask… she can be quite on and off with that, and she doesn’t like wearing one… especially with her OCD” (Young carer nine)*.

Conversely, when it was other individuals in the family unit not following the rules, this was often because they didn’t believe in COVID-19 or they believed that they were at low risk of catching the virus. In such instances, young carers reported talking to their family members directly about this, which often resulted in heated and personal arguments:

*“[It causes] very, very heated arguments…I begin talking to her about, okay, you shouldn’t be going to your auntie’s house, you should be keeping to yourself right now, you need to be at home, …she starts going, you know what, I regret having you.” (Young carer 13)*.

In such instances, participants reported feeling avoidant with family members when it came to discussing the rules to try and keep the peace, particularly as they were often stuck at home with nowhere else to go:

*“I think I’ve tried to have discussions about it, but it just turns out to be an argument, so I don’t really bother very much anymore…I try to avoid the subject” (Young carer 11)*.

### Theme 2: changes to and loss of routine

The pandemic brought huge changes to the day-to-day routines of individuals in the UK. For young carers, this meant a loss to some important aspects of their day such as time at school which provided both routine and a break from caring. This also coincided with a loss of support from services as restrictions were bought in, which resulted in an increase in caring responsibilities.

### Loss of day-to-day routine afforded via education

For many young carers aged between 11 and 16, the routine provided by school was a welcome part of the day, allowing them to focus on themselves, their education and provide some respite from caring responsibilities. However, the scramble for teaching staff to adapt lessons to online formats during the pandemic often left ‘gaps’ in the school day and removed the structures that many young carers relied on:

*“So when lockdown happened and I had nothing to revise for… no studying… I’m just left with this endless void of time” (Young carer 13)*.

### Increased caring responsibilities due to lack of support

The majority of young carers described how the pandemic increased their caring responsibilities and the burden associated with caring. The dual set of circumstances of not having access to previously available sources of support (see theme three) and not being able to go to school, work, see friends or do extra-curricular activities meant that young carers felt obligated in taking on additional responsibilities to secure the needs of the person they cared for. Many young carers reported this as challenging:

*“It was quite full-on, because we were at home 24 − 7, so (I) just had a lot more responsibilities in the house…I had to cook more often, I had to clean more often. Where, before, we could go to the community centre and do community activities” (Young carer four)*.

For some, this increase in caring responsibilities meant clashes with other priorities such as their education. Instead of having protected time away from home to study, being stuck at home and therefore constantly available resulted in taking on additional tasks and disrupted learning which caused individuals to fall behind:

*“I think it was just difficult because I couldn’t balance school and having to look after my mum at the same time….so I just had to focus more on my mum rather than schoolwork” (Young carer 11)*.

For others, being stuck at home and having increased caring responsibilities meant that for the first time, they became aware of the severity of health problems the person they cared for was experiencing. Seeing these problems ‘head on’ was described as frightening due to the young person having to confront how frail or vulnerable their loved one was without access to support:

*“His feeding, for example, I’ve noticed that he actually chokes a lot more than usual… Partly when he has difficulties breathing, and that scares us, because he has an DNR, so it’s do not resuscitate, but we get scared, if something were to happen, we can’t do anything about it. I never used to see that before because I was always at uni during his feeding” (Young carer one)*.

For other participants, whilst their caring responsibilities increased, not having to go to school or University or being afforded more adaptable education or work schedules brought a welcome degree of flexibility to their routine. This was often the case when they were not solely in charge of care, or when the individual they cared for had greater independence which left more time to carry out additional responsibilities and resulted in fewer stressors:

*“It’s been quite good [pandemic changes], actually, because I’m still able to do my caring responsibilities… I take regular breaks and I take my lunch break every day…I just do some cooking, some cleaning and anything else” (Young carer four)*.

### Loss of self-care routines and practices

All participants spoke about needing their own time and space before the pandemic as a form of self-care, to help manage the demands of being a young carer. This varied in nature but included hobbies, sports and creative outlets and often involved being outside the home. As restrictions were bought in, this limited young carers ability to engage in certain activities that supported their mental health such as sports or going to the gym:

*“When I stressed out… I’ll go to the gym, go for a walk, have a workout,… but during lockdown, everything was closed, so I didn’t get that chance” (Young carer one)*.

Not being able to leave the house due to pandemic restrictions and increased caring responsibilities meant that young carers were unable to find the time to cope and decompress. This led to feelings of isolation, low mood, as well as feeling trapped and overwhelmed:

*“Sometimes when I’d have mood swings they would be triggered by anyone just coming in my personal space when I’m trying to have that alone time” (Young carer three)*.

### Theme 3: reduced access to pre-pandemic informal and formal support structures

To help manage caring responsibilities before the pandemic, many young carers outlined that they relied on both formal sources of support such as healthcare services and young carer organisations and informal sources of support such as family members. Rules around how individuals were allowed to interact meant that these support structures drastically changed, with limited contact from wider family members, reduced health and social care support and limited emotional support provided by schools and friends. Some third sector agencies did however provide additional or new support via financial incentives and food packages.

#### Limited in-person contact with the wider family

Prior to the pandemic, some young carers were supported in their roles by other members of their extended family who were able to help by ‘sharing the load’ of caring responsibilities. However, as the rules and guidelines limited contact between different households this meant that many young carers lost this additional support and had to take on extra responsibilities:

*“When I used to go to Uni, I had my uncle or someone at home, so they would do some of the things,… I don’t get that extra help [now], because they’re in their house during lockdown.” (Young carer one)*.

Not having this support left young carers feeling overwhelmed and stressed as they were often the only individual managing their family member’s care and felt they were always on call:

*“It was mentally and physically draining, because you’re not just having to think about doing this for an hour, it’s 24 − 7” (Young carer one)*.

#### Limited contact with friends for emotional support

To help manage the demands of caring, young carers also spoke about the importance of friends as a source of support. Having friends and being part of friendship groups meant they could enjoy similar experiences to their peers such as socialising and going to the movies. The pandemic restrictions meant that most of these activities were limited to digital or video format. This resulted in friends spending less time together as the pandemic took its toll on people’s routines and health:

*“Friends that I already knew, we saw each other much less, because everyone had a lot of things on their plate“(Young carer two)*.

Whilst most young carers acknowledged the fragmenting or breakdown of friendships as a negative outcome of the pandemic and experienced this as a loss of an important lifeline beyond caring, for others, these feelings were tempered by an acknowledgment and realisation that either they had changed and were not the same person as before the pandemic, or that the people they classed as friends were only due to proximity rather than shared values or hobbies:

*“we all noticed that mostly we had nothing in common, over the lockdown we’d all changed” (Young carer six)*.

Following this realisation, young carers stressed the importance of being around people that ‘mattered’ or who were more aligned to their own interests.

#### (Limited) wellbeing support via educational pathways

Educational settings not only provided routine and structure but also acted as a conduit for accessing mental health support from staff, as well as more formal support from child and adolescent mental health services. Some younger carers reported that there was less support available than before the pandemic due to the system being overloaded, with staff juggling changes to learning and an increased need for support as the pandemic worsened the mental health of young people more generally:

*“Inside of school there were counsellors on hand… ready if you needed them, and it was just helpful sometimes to have someone to go and talk to. But lots of that has been put on pause during the COVID-19 situation” (Young carer 14)*.

When these support structures were not in place, young carers reported feeling anxious, low, depressed, struggling to cope and let down. However, for young adult carers in further education [18–24], or for those whose young carer status was known by teachers in statutory education, support was more readily available:

*“I had a teacher that was set to me because I was a young carer. They would email me once a week just to ask how I was” (Young carer six)*.

Whilst this support was welcomed, for those that received it there was a feeling that it was limited in scope or that it came too late, either because school staff had competing priorities due to the pandemic or because the young carer was no longer eligible as they were leaving secondary school or completing further education:

*[It took] two or three months…by that time, it was too late, because then the lady was like, you’re not going to be a student anymore, because I will be graduating soon” (Young carer nine)*.

#### Limited health and social care services to support young carers

Prior to the pandemic, many young carers relied on the support of different health and social care workers to help facilitate the care of the person they looked after. This included care workers who would help with personal hygiene and daily living as well as nurses and other healthcare professionals. As restrictions and social distancing measures were put in place, this meant that some home visits were stopped, scaled back, or used only to provide essential care rather than additional support:

*“So, yes, the support had been minimised a bit…because there are other priorities, or it’s because of lockdown, they’re trying to minimise them coming into our house or us going to the hospital” (Young carer one)*.

Social distancing restrictions also meant that in-person health and social care appointments for the young person and the person they looked after were cancelled or rescheduled unless perceived as an emergency:

*“Yes, appointments have been less frequent. Non-essential ones like the dentist, unless you had an urgent problem, that was put on pause” (Young carer five)*.

Whilst there was an acknowledgement by young carers that health and social care staff were doing the best they could, the use of a remote appointment system was experienced as inappropriate in some instances, particularly for assessments of physical health complaints requiring a physical examination:

*“The issue of needing to see someone, so they could assess her feet and her legs, you can’t really do that over the phone” (Young carer seven)*.

This caused increased worry and was experienced as substandard care with health needs not properly addressed. Moreover, in many instances, young carers felt dismayed and hopeless as not getting the necessary treatment support resulted in their family member deteriorating and them being left to cope with this on their own:

*“Things took longer than needed with mum’s health…I had to cope with her mental health being bad, in order to get support. And then, once the support started, it took quite a while” (Young carer two)*.

#### Local authority and non-statutory bodies ‘filling the gap’

Whilst support from family and friends and some formal services was unavailable, or not as accessible due to pandemic related restrictions, services provided by local authorities and voluntary organisations provided additional or adapted support to participants. Often this was possible via special emergency allocations made accessible for vulnerable groups from local government and charities:

*“They’ve [young carers] been getting all the different supplies for activities from us… Whenever we’ve been getting funding we’ve been able to give what we think they need” (Service provider seven)*.

In the first half of the pandemic, services reported providing financial support to meet the practical needs of young carers and family members for essentials such as food, bedding and clothing. However, services reported being unable to provide cash directly and instead issued shopping vouchers for major supermarkets:

*We had quite a number of financial support requests…we were issuing out Tesco vouchers to try and alleviate some of that financial pressure (Service provider three)*.

Young carers reflected that having this financial support was a lifeline, particularly for those who were furloughed or out of work coupled with being stuck at home which led to increased food and utility costs:

*“So, I think I got £400 from the carers centre. That really supported me, [Name], in terms of getting basic food and drink, but also basic clothing items, even and just some bedding materials” (Young carer four)*.

In addition to redeemable vouchers, local authorities and other third sector organisations also provided food packages to young carers and their families so that they were not unduly exposing themselves and indirectly their loved ones to the COVID-19 virus.

*“the council used to drop off food to more vulnerable people…stuff like perishables and more snacks, we would allocate one person to get them” (Young carer eight)*.

On top of the essentials to survive, support came in other formats too. One support provided by third sector organisations was to provide electronics and wifi to enable young carers to study online and access online support groups:

*“half of their houses didn’t have wifi in them, so we were having to go and buy dongles for them…to be able to come and do a Zoom. When the schools were taking the Chromebooks back…we then applied for so many and then I was going around giving them to the families that were most at need” (Service provider eight)*.

Third sector organisations also provided emotional and social support for participants during the pandemic. In its initial stages this was only possible via videocall. This often provided a dual purpose, allowing young carers a forum to support one another and knowing that individuals on the call were in the exact same situation as them:

*“Even though it was online, it just felt nice to have a support network and a group of young carers, like myself, who we could share experiences and I didn’t feel as isolated in my own struggles” (Young carer nine)*.

However, some young carers found it challenging to engage in these online activities due to Zoom fatigue and being ‘stuck’ in the environment where they performed their caregiving activities which meant that it was sometimes seen as another task, rather than a social activity. Low attendance at online support sessions set up by local organisations for young carers was also acknowledged by service providers. Similar to reports from young carers, there was a feeling that ‘zoom fatigue’ from individuals being online all day for educational purposes was contributing to this:

*I know that one of the reasons that the young people didn’t want to engage with us so much was because it was just more screen time (Service provider eight)*.

As pandemic restrictions eased, young carers spoke about appreciating opportunities to meet in person in socially distanced ways facilitated by young carer organisations. This helped them to feel more connected to others in a face-to-face format:

*“I really enjoy the physical sessions that’s now, because it’s just you can see the whole body, you can see the whole emotion, much better, more clearer… so I just prefer that much more. Now, I’m attending every single week” (Young carer eight)*.

### Theme 4: better understanding of inner resilience and goals

When reflecting on the pandemic and what they had learnt, young carers spoke about how it had taught them about their own capabilities, resolve and resilience in the face of adversity, as well as allowed them to prioritise areas of importance for them. For some young carers, their increased resolve and capabilities were related to how they managed to adapt to the increased demands of caring during the pandemic, often with less support than before the pandemic:

*“I do feel a bit happier now, because…I felt like, although I had more responsibilities, I managed to fulfil them. So that sense of fulfilment and achievement that I felt, although I didn’t receive that much external support” (Young carer four)*.

For others, the pandemic provided them with the opportunity to reflect on what was important to them and to implement strategies to achieve goals in relation to this. Key areas that young carers discussed here related to strategies to support their own mental health and wellbeing, as well as their education:

*“So, my school chose not to give any materials to work on out, so in that six months, I just created a plan, a timetable, where I would work every single day in revising. It was a bit more independent on myself” (Young carer eight)*.

## Discussion

We aimed to explore the impact of the pandemic and associated restrictions on mental health, wellbeing and access to support for young carers living in the UK and make suggestions for future service provision. We also aimed to understand how young carers could be better supported in future health emergencies. Whilst the pandemic had negative consequences for many young people [24], young carers have been disproportionally affected, with research highlighting increased mental health difficulties and lowered wellbeing when compared to peers with non-caring responsibilities [27, 28].

### Supporting young carers mental health and emotional wellbeing – the role of schools and other agencies

In keeping with previous findings, many young carers spoke about the importance of routine as this helped them manage their daily caring responsibilities [29]. School was seen as an important part of this routine as it provided a form of self-care and respite [29].However, a novel finding from this study was that participants also described how school provided a place to access emotional support via school staff and links into other agencies. As schools went into lockdown and staff became overwhelmed with tasks such as adapting lessons for online teaching, this meant that young carers lost or had limited access to this emotional support pathway. This, combined with losing other sources of support may have contributed to the increases in mental health difficulties and lowered wellbeing observed in other studies [27, 28]. To support young carers in future, schools should actively enquire with students each year to understand which pupils may be classified as young carers. However, as some young people do not know they fall under this classification [36], this should be handled in a sensitive way by form tutors or pastoral staff equipped with the knowledge and skills to be able to do so. New initiatives such as ‘Young Carers in Schools’ [37] may provide a useful framework to do this, with guidance on how to implement best practice to identify and support young carers in this setting. In a 2018 evaluation of this programme involving 115 schools, 94% of schools reported that their staff were more likely to be able to identify young carers and know what to do once they were identified [38]. Once identified, policies and procedures for protected time with young carers should be implemented to secure both their emotional wellbeing and support their learning both in and out of health emergencies. Given the limited time within the school day coupled with their caring responsibilities, possible considerations may include greater 1–1 support provided by school staff during school time, modified timetables and partnering with young carer organisations who can also provide expertise and facilitate education and emotional support. Trialling such approaches have received positive responses, with young carers improving their attendance and reporting that the support helped them emotionally [39].

An important finding identified from participant interviews in this study was the additional and enhanced forms of support provided by other agencies, predominantly local authorities and third sector organisations. Participants reported that these organisations not only provided emotional and wellbeing support but acted as a conduit to address their basic physiological and safety needs and facilitated access to resources such as funds and food parcels. This meant that for some young carers they were able to concentrate on other priorities such as additional caring responsibilities or their education. Prior to the COVID-19 pandemic, young carers reported that interventions by such agencies tended to predominantly focus on emotional support activities [40] however this research has highlighted these services’ dynamic ability to adapt and support this group during a time of uncertainty. Given the importance of local authorities and third sector organisations ability to step in to support this group when other services were overwhelmed, Governments should consider ring fencing additional funds specifically for supporting the physical and emotional wellbeing of young carers. This aligns with calls from charities and national bodies [41]and will also help to address the lack of educational and mental health parity when compared to their peers without caring responsibilities [16].

### Supporting young carers mental health and emotional wellbeing – the role of friendships

Whilst schools were one source of emotional support, many young carers reported peer support and friendship groups as important sources of emotional support as they allowed time for respite and to engage in similar activities to their peers. Practically, the closure of schools and pandemic related restrictions such as lockdowns meant that young carers were only able to see their friends digitally. For some, this resulted in the fragmenting of friendship groups whose size did not easily adapt to these digital interactions or disengagement from friendship groups due to zoom fatigue. For others, more caring responsibilities meant they had less time to engage with friends and peers. Results from a previous cohort study found that during the pandemic, increased distress and lower mental wellbeing were identified in young carers and driven by aspects such as low social support and feelings of isolation [28]. Given the importance placed on friendship groups for young carers, our findings help shed further light on specific reasons for the breakdown of these social support structures. To protect young carers during future health emergencies, a solution may be to allow local authorities or third sector organisations to facilitate or host in-person support bubbles specifically for those with caring responsibilities, particularly as those we interviewed followed the rules because they did not want to put those they cared for at risk. This level of adherence to the rules suggests that young carers would be less likely to spread the virus within those support bubbles. Another solution would be to facilitate socially distanced outdoor activities between young carers. Such recommendations may be supported by qualitative findings that young carers reported better mental health when they were able to meet peers and friends in person and in socially distanced ways when restrictions eased [31].

### Supporting young carers and the people they look after

Whilst previous research has reported an increase in distress for individuals lacking capacity around the enforcement of COVID-19 rules, the focus has been on adults looking after either young people or adults that lack capacity [42]. Our research sheds insight into the distress this caused young carers who may not have had the necessary tools or authority to be able to reinforce these rules with those they looked after, increasing the possibility of them being exposed to a potentially fatal virus. Simply providing general information to people to change their behaviour is often unsuccessful [42], so in future health emergencies, the Government, as well as local and regional public health experts should draw on the support of public health experts to provide targeted messaging to young carers and their families, as well as practical strategies to support adherence to guidelines. Targeted messages and support strategies may wish to draw on CBT-informed and easy-to-understand communications [43] for those with cognitive or mental health difficulties. Enlisting the help of local organisations who support relevant populations and whom individuals trust should also be considered as a strategy as this has been shown to increase adherence to the guidelines [44].

Our sample was ethnically diverse, with nearly three quarters (71%) being from minority ethnic backgrounds. Whilst this proportion is higher than the national average [45] it provides important insights into underserved groups who are young carers and has implications on how these individuals can be supported. Recent reports have identified that young carers from Asian backgrounds are more likely to be unhappy across most aspects of life when compared to White young carers [46], with particularly high disparities around happiness with their education progress. This may be due the cultural importance Asian families place on educational attainment [47]which is more difficult to achieve due to caring responsibilities [48]. Given the importance of some specialist third sector organisations in supporting and building strong relationships with young carers, consideration should be given to developing pathways for young carers into services, with special attention being paid to young carers with intersectional characteristics such as those who are from minority backgrounds. This is particularly important as currently pathways into mental health services for minority ethnic young people are more likely to be from youth justice or social care settings and therefore compulsory in nature [49]which may cause stigma, remove choice and empowerment and lead to worse health outcomes [50].

### Strengths and limitations

This study builds on initial research conducted with young carers at the start of the pandemic by allowing participants to reflect on later stages as restrictions eased and guidelines changed. Our research contributes an in-depth picture of the impact of the pandemic for young carers, exploring a range of issues with participants and provides recommendations that can be carried forward to protect this group in future health emergencies. Our sample was ethnically diverse and represented a range of caring responsibilities and caring relationships. However, whilst a range of viewpoints were sought, this study relied on a convenience sample largely composed of females and those of Asian heritage, thus viewpoints from certain demographics may be missing or under-represented (e.g. young carers from other minority ethnic backgrounds or younger carers of primary school age). Moreover, we used remote interviewing techniques which may have excluded some groups with limited online access or those who were reporting ‘Zoom fatigue’.

### Future research

Future research should focus on working with young carers, their families and organisations who support them to explore how best to implement these recommendations into practice. Moreover, given that these recommendations were developed through researcher interpretations of interviews with young carers, future research should also explore directly with young carers other strategies which may be helpful in supporting them in future health emergencies. To translate findings into practice, research findings should incorporate implementation science or behaviour change frameworks to understand how to adapt and enhance access to existing programmes of support and co-produce outputs with young carers for maximum reach and effectiveness. Future research should also consider perspectives from young carer populations not included in this sample to ensure findings are translatable using purposive sampling and working closely with young carer organisations across the country to target specific groups. It should also consider the views of family members looked after by young carers, to understand their experiences during health emergencies, as well as their views on how to support both themselves and young carers in future. Lastly, whilst young carers in this paper discussed their mental health and how this was affected by the COVID-19 pandemic we did not objectively measure mental health symptoms as part of this study. Given that mental health difficulties are known to be more prevalent in young carers compared to non-carer peers [16], future research may wish to consider including screening measures prior to interviews. Widely used measures such as the Revised Children’s Anxiety and Depression Scale (RCADS) [51] or the Strengths and Difficulties Questionnaire [52] could be used to better understand the interplay between mental health and caring responsibilities in future health emergencies.

## Conclusions

We suggest a range of interventions that could be implemented across different systems to support young carers in future health emergencies and make sure this group is not overlooked in future. From a Governmental perspective, ministers should consider guidelines and provisions to allow for support bubbles involving groups of young carers, so that they are able to provide emotional support to one-another, as well as ring fenced funding specifically for this cohort so that local authorities and third sector organisations can provide physiological, social and emotional support. Third sector organisations and local authorities should develop policies and guidelines based on their learning to further streamline support for young carers in future healthcare emergencies but also in a post-pandemic world. Lastly, those responsible for public health, should consider working closely with psychologists and local authorities to create targeted messaging to reinforce health promoting behaviours for those with cognitive impairments or for wider family support networks of populations vulnerable to the negative health impacts of the virus.

### Electronic supplementary material

Below is the link to the electronic supplementary material.

Supplementary Material 1

Supplementary Material 2

## Data Availability

The datasets generated and/or analysed during the current study are not publicly available, nor are they available upon request, because the dataset consist of interview transcripts that might compromise participant privacy and confidentiality due to the sensitive nature of topics discussed.

## Abbreviations

GCSE: General Certificate of Secondary Education
GP: General Practitioner
OCD: Obsessive Compulsive Disorder
UCL: University College London
UK: United Kingdom
