# Supplementary material for: The experiences and impact of the COVID-19 pandemic on young carers: practice implications and planning for future health emergencies
Source: Child Adolesc Psychiatry Ment Health. 2024 Jan 3;18:2. doi: 10.1186/s13034-023-00697-6 (PMC10765662; doi:10.1186/s13034-023-00697-6)
Supplement: Supplementary file 1 — Supplementary Material 1 [file 13034_2023_697_MOESM1_ESM.docx]

**S1: Participant demographics**

| **Participant** | **Age range** | **Gender** | **Who do they care for** |
| --- | --- | --- | --- |
| YC01 | 18-21 | Female | Multiple family members |
| YC02 | 22-24 | Female | Parent |
| YC03 | 22-24 | Female | Parent |
| YC04 | 22-24 | Male | Parent |
| YC05 | 22-24 | Male | Parent |
| YC06 | 14-17 | Female | Grandparent |
| YC07 | 18-21 | Female | Parent |
| YC08 | 18-21 | Male | Grandparent |
| YC09 | 18-21 | Female | Parent |
| YC10 | 22-24 | Female | Multiple family members |
| YC11 | 14-17 | Female | Parent |
| YC12 | 14-17 | Female | Sibling |
| YC13 | 14-17 | Female | Parent |
| YC14 | 14-17 | Male | Multiple family members |

**S2: Reflexive statement from the primary researcher**

When starting this analysis, I knew relatively little about the experiences of young carers, though I had a decade of research experience in child and youth mental health with an emphasis on community and school settings. Given recent advances in intersectionality and health inequalities, I suspected that this group would have fared less well than some of their peers during the pandemic. Whilst this was in part true, it paints a one-sided picture, as some young carers reported benefits in better managing their care responsibilities and this was reflected on during supervision and in group meetings. I also, having conducted much research in schools and seeing the benefit of integrating mental health support and schools, suspected that they would have been an institution which was better able to support the needs of this population. As themes were developed, I was slightly surprised that there were not more safeguards made for young carers by schools during the pandemic. However, on reflection, this must also have been an incredibly difficult time for schools and staff who were under incredible strain in adapting education to a virtual format. To try and make sure my recommendations were balanced considering these assumptions, I consulted three school pastoral staff who helped shaped recommendations which they believed were implementable for young carers in schools.
